# Clinical and Functional Effects of Rehabilitation of Patients after COVID-19 Infection

**DOI:** 10.3390/jcm13113257

**Published:** 2024-05-31

**Authors:** Zofia Dzięcioł-Anikiej, Anna Kuryliszyn-Moskal, Monika Pociene, Janusz Dzięcioł, Agnieszka Dakowicz, Amanda Kostro

**Affiliations:** 1 Department of Rehabilitation, Faculty of Health Sciences, Medical University of Białystok, Skłodowskiej-Curie 7A Street, 15-096 Białystok, Poland; anna.kuryliszyn-moskal@umb.edu.pl (A.K.-M.); agnieszka.dakowicz@umb.edu.pl (A.D.); amanda.kostro@umb.edu.pl (A.K.); 2 Department of Physiotherapy and Beauty Therapy, Klaipedos Valstybine Kolegija, 91274 Klaipeda, Lithuania; m.pociene@kvk.lt; 3 Department of Human Anatomy, Faculty of Medicine, Medical University of Bialystok, Mickiewicza 2A Street, 15-230 Białystok, Poland; janusz.dzieciol@umb.edu.pl

**Keywords:** COVID-19, rehabilitation, posturography

## Abstract

**Background/Objectives:** The most common post-acute consequences of SARS-CoV-2 include lung dysfunction, the impairment of cognitive functions and mental health, as well as the impairment of the musculoskeletal system in the form of fatigue and muscle weakness. Post-COVID-19 patients often experience impaired balance and reduced physical capacity. It is important to implement a rehabilitation program that eliminates the side effects of COVID-19 and allows for significant improvement in the patient’s functionality. The aim of our study was to assess patient functionality after a 6-week rehabilitation program on balance, foot pressure distribution, and physical capacity in post-COVID-19 patients. **Methods:** The clinical study group consisted of 53 people 3 months after COVID-19 infection, confirmed by a positive PCR test. Exclusion from the study included people with comorbidities that impaired balance and gait. The patients underwent a posturographic assessment—Romberg test, a baropodometric assessment—static and dynamic, and a performance assessment—a 6 min walk test determining shortness of breath on the mMRC scale, blood pressure, heart rate, and saturation. Patients participated in rehabilitation until the sixth week, after which they were assessed again. Comparisons were made using IBM SPSS Statistics 27.0 software using the Wilcoxon pairwise order test, at a significance level of *p* < 0.05. **Results:** The result of the postural control assessment showed an improvement in the ability to maintain the centre of gravity in terms of the foot support area—statistical decreases were observed in the ellipse area, from 745.28 mm^2^ to 453.52 mm^2^ (*p* = 0.009), as well as maximum (from 3133.5 gr/cm^2^ to 2994.2 gr/cm^2^; *p* = 0.065) and average load on the left foot (from 1010.1 gr/cm^2^ to 969.38 gr/cm^2^; *p* = 0.028). In the 6 min walk test before and after exercise, the heart rate decreased after the therapy (shortness of breath on the mMRC scale also decreased from 79.12 to 74.95). This means that patients achieved better physical fitness and efficiency. **Conclusions:** Rehabilitation significantly improved balance, as measured by a decrease in ellipse area during the Romberg test.

## 1. Introduction

Falling sick with COVID-19 results in a number of health hazards not only at the moment of infection but also in long-term sequelae after recovery [1,2]. The most frequently observed impairments include impaired respiratory function, cognitive functions, and mental functions as well as muscular functions manifesting themselves through muscle fatigue and weakness, which matters as far as a patient’s daily life is concerned [3]. Furthermore, other research indicates that it takes seven months for many patients to achieve full recovery, and a failure to do so causes persisting symptoms involving multiple-organ impairment such as impaired systemic functions, cognitive function, or neurological function [4]. Reduced physical activity or the lack of it due to a long-term hospitalisation are presumed to cause multiple-organ impairment in the case of individuals suffering from persisting COVID-19 symptoms or post-COVID-19 conditions, which may adversely impact the postural control system, for instance, while rising to an erect posture. The research indicates that post-COVID-19-hospitalised patients show impaired visual and somatosensory cognitive function in terms of erect posture [5]. Furthermore, other sources confirm the fact that post-COVID-19 patients obtain lower postural control test results as compared to the control group [6] and differ in terms of the foot pressure distribution as far as baropodometric assessment is concerned [7]. The neuromuscular system involves muscles and the nervous system to control movements of the body and maintain postural balance, and COVID-19, impairing that system, may result in long-term sequelae affecting the postural control system [8]. To prevent unfavourable post-COVID-19 symptoms, the available literature suggests implementing a hospital rehabilitation program. The results of research on the use of rehabilitation in people who have suffered from COVID-19 and were hospitalized in the ICU show an improvement in balance, muscle fitness, and strength, as well as the functioning of the respiratory system, which improves the quality of everyday life [9].

## 2. Purpose

The aim of this study was to conduct the functional assessment of static and dynamic baropodometric and postural control parameters and the physical capacity of patients undergoing 6-week outpatient rehabilitation, including physical efficiency training sessions as well as respiratory and multiple-organ function improvement exercises. The aim of our study was to assess the patient’s functionality after rehabilitation.

## 3. Methods

The clinical study was conducted at the Rehabilitation Clinic under the consent issued by the Bioethical Commission no APK.002.51.2022 at the Medical University of Białystok. The research was conducted from January 2022 to January 2023. Patients were assessed among people who were referred to a Rehabilitation Clinic for post-COVID rehabilitation. Patients participating in the study were present at each rehabilitation session. The qualified eligible patients were diagnosed at least 3 months prior to the study. At the moment of the study, no post-SARS-CoV-2 side effects were reported. Exclusion from the study included individuals with underlying conditions impairing postural control and gait such as obesity, neuromuscular disorders, neurological deficits or sensory disorders, spinal or lower limb surgery, or injury impairing stability, for instance, 2nd–3rd degree ankle joint sprain as well as the use of crutches, walking sticks, walking frames, etc., for the purpose of daily activities. The exclusion criteria also included smokers without any previous diseases or operations within internal organs such as the respiratory, cardiovascular, and digestive systems. All patients declared that their occupations did not involve physical labour.

During qualification, all patients reported dominance on the right side of both the upper and lower limbs.

**Figure 1:**
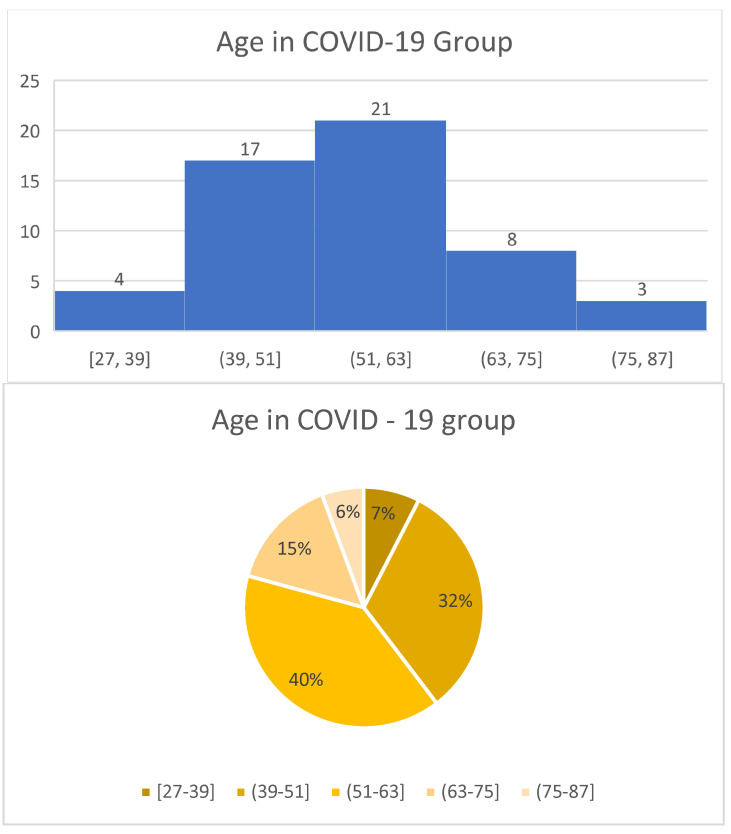
Age in COVID-19 Group.

Each of the patients issued informed consent to take part in the study and was notified of the effects and course of the study. The study group comprised 53 post-COVID-19 (30 women and 23 men) patients whose diagnosis had been confirmed by the clinical symptom-based examination results and positive PCR test—covering the polymerisation chain reaction (Table 1, Figure 1).

The patients suffered from moderate symptoms of the disease, the prevailing symptoms having been cough, fever, muscle pain, loss of the sense of smell, and taste disorders.

The patients underwent functional assessments to the extent of an interview, the assessment of postural control to maintain balance while standing by means of Romberg’s test, static and dynamic baropodometric parameters, as well as physical efficiency testing including blood pressure, pulse, and saturation measurement by means of a 6 min walk test. Furthermore, dyspnoea was assessed in terms of the mMRC Scale. Next, for a period of 6 weeks, the patients performed personalised training workout programmes, respiratory exercises, and multiple-organ function improvement exercises, after which they underwent a functional reassessment test procedure.

**Figure 2:**
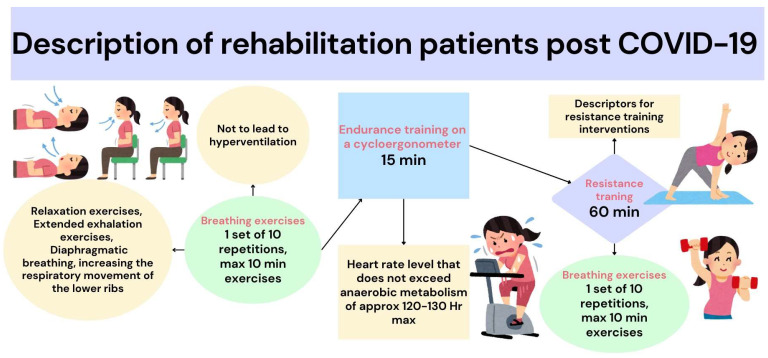
Description of rehabilitation patients post COVID-19.

The training included resistance exercises for the upper and lower limbs, taking into account the movements of flexion and extension, abduction and adduction, as well as internal and external rotation. This was performed in two series, taking into account the number of repetitions at 70% load and the feeling of fatigue on the RPE scale up to 7. Included breathing exercises were based on relaxation exercises, extended exhalation exercises, diaphragmatic breathing exercises, and exercises increasing the respiratory movement of the lower ribs, and they were performed in two series—at the beginning and at the end of the therapy, with 10 repetitions each. So as not to lead to the patients’ hyperventilation, endurance training was performed on a cycloergonometer at a heart rate level that does not exceed an anaerobic metabolism of approx. 120–130 Hr max. The training was conducted on the basis of our own experience based on clinical work. Each patient was treated individually under the supervision of a therapist through a one-on-one method. The duration of the entire training session is 60 min of resistance exercises, 15 min on a cycloergometer, and 10 min of breathing. Resistance exercises were performed simultaneously on both limbs. (Figure 2. Description of rehabilitation patients post COVID-19).

The therapy was conducted by a qualified therapist under their constant supervision, at the same time and place, using the same device. Before and immediately after therapy, patients reported possible side effects and increased shortness of breath (above 1 on the mMRC scale). The exercises were based on the patient’s subjective feelings (Table 2).

Postural and baropodometric stability were assessed on the FreeMED Base tensometric platform by means of the dynamic, static, and postural control analysis based on a 1 min Romberg’s test with eyes closed and open. The patients were examined by means of the same device, in the same ambient conditions, at the time of a day, and by the same person performing the measurements. The measurements were analysed by means of the Free Step software version 1.3.5. The dynamic analysis involved each of the individuals under analysis, who walked a specific and equal distance on the tensometric platform while the foot pressure was being measured. Within the framework of the dynamic analysis, the right and left foot parameters were compared, namely, foot trace length (mm), gait length line (mm), foot surface (mm^2^), as well as the maximum and mean load (g/cm^2^). On the other hand, the static analysis encompassed the measurement of the foot pressure for 5 s while standing in the upright position with upper limbs. The following parameters were analysed: foot surface (cm^2^), foot load (%), and maximum and mean foot load (g/cm^2^). The postural control assessment entailed the continual measurement of the centre of the foot pressure (COP), the displacement of which enables us to obtain the balance details. The following parameters were compared: swing length (mm), surface area (mm^2^), speed (mm/s), and the length of the minimum and maximum swing of the patient’s centre of gravity. The 6 min walk test entailed the pre- and post-test measurement of the following parameters: systolic and diastolic blood pressure, pulse, saturation, and dyspnoea in terms of the mMRC Scale. The results obtained before and after the rehabilitation were compared. The research did not include comparisons with a control group. Only post-COVID-19 patients were assessed.

All the variables under study were quantified or ordinal. They were compared within independent groups by means of the Wilcoxon signed-rank test. All of the computations were conducted by means of the IBM SPSS Statistics 27.0 software. The statistical hypotheses were tested in terms of the statistical significance *p* < 0.05.

## 4. Results

The dynamic analysis indicated improvement—the maximum and mean left foot load in gait decreased statistically significantly, but the right foot one increased statistically insignificantly. The results showed no change with respect to the trace length, foot surface, or gait line length (Table 3). The static analysis indicated a decrease in the foot surface, and as far as the statistical significance is concerned, the whole foot surface decreased, and the left back and forefoot pressure decreased, too. Furthermore, the maximum and mean right lower limb load increased in correspondence with the previously assumed statistical significance (Table 3). The outcome of the postural control assessment indicated the capacity for improvement in maintaining the centre of gravity as far as the foot support surface is concerned. Romberg’s coefficients decreased with respect to the swings, ellipse surface, and mean speed with eyes open, and statistical significance was indicated with respect to the ellipse surface (Table 4). Saturation statistically significantly increased, and the pulse decreased throughout the course of the 6 min walk before effort. After the respiratory and physical capacity exercises, dyspnoea parameters improved in terms of the mMRC Scale—the majority of patients considered it to be at 1 and 0 after therapy. Similar results were delivered after the 6 min walk test. Pulse and dyspnoea decreased as a result of the therapy (Table 5).

## 5. Discussion

Post-COVID-19 syndrome constitutes a new clinical challenge that requires specific therapy involvement from the medical team for treatment and diagnosis purposes. SARS-CoV-2 infection symptoms may sustain for many months after a negative test or moderate course of the disease. The symptoms do not only refer to the lungs, which are most often affected in the first phase of the disease. Most of the time, the symptoms refer to the central and peripheral nervous system, manifesting themselves in peripheral neuropathy, headaches, dizziness, muscle pain, fatigue, memory loss, depression, or sleep disorders [10,11,12]. According to the research outcomes, the diaphragm, as the main respiratory muscle, also plays a number of essential physiological functions through the sense of pain, supporting stomach–oesophagus function, and finally through posture stabilisation supported by the lumbar spine, by means of the abdominal pressure that it produces. That fact may matter in the case of the rehabilitation of post-COVID-19 patients [13]. On the other hand, the balance assessment must be considered as the process integrating numerous stimuli, and the related notion of stability should be treated as the capability to regain balance and to lose it within the sphere that is controlled by means of many properly functioning systems, including the nervous and visual systems and the proper perception and integration of stimuli from the surrounding environment, for instance, proprioception. The analysis of the centre of the foot pressure is one of the sensitive balance assessment methods applied as an auxiliary statistical measure for postural control examination purposes [14]. On the other hand, in order to assess the gait and the migrating centre of gravity pressure, baropodometric assessment methods are applied that most frequently depict the differences in the diagnostics of rheumatic disease and related dysfunctions and foot deformities [15,16,17].

A number of studies indicate that the balance disorders occur in the case of post COVID-19 patients [10,17,18,19,20,21]. The provisional outcome of examinations carried out within a group of individuals who recovered from COVID-19 infection as compared to the healthy individuals examined before the pandemic indicate that past infection affects the baropodometric as well as postural control parameters [6,7]. Our study showed that patients coordinated actions aimed at balancing the centre of pressure during walking by loading the dominant side of the body (at the beginning of the study, all patients indicated that the right side of the body was dominant). This may suggest a benefit from breathing exercises as part of an assessment of balance-related postural control. A change in the foot surface, including the rear and forefoot, and the entire foot surface area may indicate an improvement in the efficiency of the foot muscles while maintaining the stability of the foot arch. After the therapy, the load on the lower limb changed—the load on the left foot decreased and the load on the right foot increased, which may indicate a beneficial effect of postural control on the movement of the centre of pressure to the benefit of the more efficient limb. The influence of the diaphragm, which is the main respiratory muscle and is activated during breathing exercises, on balance functions and postural stabilization was already confirmed in the works of Kocjan J. [13].

The study conducted by Leszczak J. et al. assessed the impact of rehabilitation in the case of patients who had recovered from a brain stroke or a brain stroke with concurrent COVID-19 infection to the extent of mobility and balance. The study was conducted by means of Time Up and Go testing as well as the Tinetti Test, confirming that hospital rehabilitation at an early stage after a brain stroke improved mobility and balance and mitigated the risk of falls amongst the patients after a brain stroke, both with the concurrent COVID-19 infection and without it [22].

Still, another study conducted by Paéz W. et al. was indicative of a significant improvement in quality of life and functional capacity as well as a reduction in mental and cognitive strain within an 8-week rehabilitation programme. The significant improvement was proven by a variety of physical capacity tests, including the 6 min walk test, 1 min sitting–rising test, dynamometry, the Tinetti Test, and the Berg Balance Test (*p* < 0.001) [23]. Accordingly, that study also indicated an improvement in the physical capacity measured by means of the 6 min walk test—the saturation and pulse parameters before the test improved, and the pulse parameters after the test improved due to the therapy that had been applied.

There are no relevant reports on the assessment of baropodometric parameters to the extent of foot pressure amongst post-COVID-19 patients after rehabilitation. However, researchers undertook the assessment of gait and respiratory function in the case of a patient who had undergone a severe COVID-19 course by means of a robot workout. The patient—a 48-year-old woman who had undergone a severe COVID-19 course—was subject to the 6-week hospital rehabilitation programme. The patient showed persisting lung function and physical capacity impairment, including gait and balance control disorders. After the training workout, the maximum inspiratory pressure and maximum exhalation pressure improved, the gait pace accelerated, and the balance control improved, too. As far as the physical efficiency was concerned, the distance covered during the 6 min walk test lengthened with concurrent pulse and saturation improvement [24]. The aforementioned study is indicative of similar results to those shown here.

An improvement in the result of the 6 min walk test was also achieved under the study conducted by Nopp S. et al. Patients improved their test results by 62.9 m (±48.2, *p* < 0.001) on average; moreover, a significant improvement was delivered with respect to dyspnoea (*p* < 0.001), fatigue (*p* < 0.001) and quality of life (*p* < 0.001). The lung function parameters (the expiratory volume per 1s, the lung diffusion capacity, and the inspiratory pressure increased) improved throughout the rehabilitation course. This result allows us plausibly to state that in patients who underwent a severe COVID-19 course, their physical efficiency, functional condition, dyspnoea, fatigue, and quality of life improved after 6 weeks of the personalised interdisciplinary respiratory rehabilitation workout programme [25]. In our study, the patients also achieved a better physical capacity after outpatient rehabilitation including respiratory rehabilitation.

The pandemic period contributed to the occurrence of many functional disorders throughout the body.

Restrictive measures against the effects of COVID-19 taken by the governments of many countries were aimed at eliminating the spread of the pandemic, which resulted in a reduction in the number of people suffering from otitis media, which is confirmed by the work of G. Iannella and co-authors [26].

This is why comprehensive preventive measures, including systemic rehabilitation, are so important.

## 6. Conclusions

Physical and respiratory capacity rehabilitation was very much beneficial for patients who underwent SARS-CoV-2 infection in terms of saturation, pulse, and dyspnoea.

Notwithstanding the lack of a training workout focusing on balance control, gait, and postural stability, a positive impact was provisionally confirmed to the extent of gait and balance control amongst post-COVID-19 patients who had undergone physical capacity and respiratory rehabilitation—an amazing effect was observed on the centre of gravity migration function while walking, and foot arch shape was achieved as a result of respiratory and physical capacity therapy, notwithstanding the lack of exercises focusing on foot muscle functional capacity.

These results may confirm a key role of the diaphragm as a stabiliser muscle contributing to static and dynamic function—gait—requiring further research.

Limitations, Recommendations, and Generalizations: The study conducted had both limitations and benefits. Limitations include the fact that the results of posturgraphic and functional tests may be influenced by the patient’s resting state or mood, which may reduce test values. Future studies should consider including an additional survey assessing the patient’s mood and wakefulness. Additionally, patients’ levels of physical activity may also impact their ability to maintain balance, which should be considered in further research. The presented posturographic and clinical assessment can be used both in the offices of primary care physicians and physiotherapists to detect balance disorders in people post-COVID-19. The research did not include comparisons with a control group. Only post-COVID-19 patients were assessed.

## Figures and Tables

**Table 1:** Demographic description of COVID-19 group.

		BMI				Age			
Gender	n	Mean	Median	Min	Max	Mean	Median	Min	Max
Woman	30	28.28	27.68	21.26	40.75	54.23	55.00	27.00	85.00
Man	23	29.83	29.83	29.83	29.83	54.04	55.00	30.00	86.00

**Table 2:** Descriptors for resistance training interventions.

Descriptor	Example
Load magnitude	70% 1RM
Maximal number of repetitions based on RPE (rating of perceived exertion) scale of 7	10
Number of sets	2
Rest in between sets (seconds or minutes)	1 min
Number of exercise interventions (per day or week)	3 per week
Duration of the experimental period (days or weeks)	6 weeks
Fractional and temporal distribution of the contraction modes per repetition and duration (seconds) of one repetition	4 s shortening, 1 s isometric, 4 s lengthening
Rest in between repetitions (seconds or minutes)	None
Range of motion	100%
Recovery time in between exercise sessions (hours or days)	48 h
Anatomical definition of the exercise	Yes, must be included

**Table 3:** Dynamic and static analysis of the patients before and after the therapy.

			Mean	Standard Deviation	Minimum	Q1	Median	Q3	Maximum	*p*
		
Dynamic Analysis	Trace Length LF mm	Before Therapy	252.39	21.75	220.19	232.82	249.23	268.73	308.18	0.072
		After Therapy	250.69	23.98	210.17	237.64	245.61	261.74	354.61	
	Trace Length RF mm	Before Therapy	254.79	26.46	194.08	234.76	250.57	276.69	341.97	0.566
		After Therapy	252.77	24.38	210.73	234.24	250.17	270.01	309.57	
	Gait Line Length LF mm	Before Therapy	213.84	30.64	60.21	204	217.83	235.28	255.05	0.695
		After Therapy	218.24	19.44	165.08	205.79	217.83	232.11	275.39	
	Gait Line Length RF mm	Before Therapy	217.63	25.6	147.14	201.15	215.52	240.05	270.6	0.11
		After Therapy	214.47	22.3	165	200.88	217.08	230.68	267	
	Surface LF cm^2^	Before Therapy	117.68	17.67	84	104	116	129.5	153	0.678
		After Therapy	117.66	20.48	81	103	120	131.5	175	
	Surface RF cm^2^	Before Therapy	119.15	20.52	82	101	119	131.5	167	0.832
		After Therapy	118.43	23.26	80	101	116	129.5	195	
	Max Load LF gr/cm^2^	Before Therapy	3133.5	442.87	2108	2804	3100	3384	4172	0.005
		After Therapy	2944.2	381.1	2200	2686	2948	3148	4068	
	Max Load RF gr/cm^2^	Before Therapy	3021.4	462.36	2188	2698	2944	3344	4684	0.175
		After Therapy	3536.8	3226.72	1928	2846	3152	3370	26382	
	Mean Load LF gr/cm^2^	Before Therapy	1010.1	145.67	755	888	1003	1090.5	1355	0.028
		After Therapy	969.38	126.75	722	871	982	1046.5	1365	
	Mean Load RF gr/cm^2^	Before Therapy	963.36	164.29	664	843.5	978	1067	1490	0.236
		After Therapy	1004	163.5	649	928.5	1011	1075	1476	
Static Analysis	Surface LF cm^2^	Before Therapy	80.98	22.79	34	65	79	94	128	0.01
		After Therapy	75.06	18.89	37	61.5	74	86	131	
	Surface RF cm^2^	Before Therapy	87.11	24.83	45	65	88	106	154	0.653
		After Therapy	85.51	22.61	47	69	84	101	153	
	Forefoot Surface LF cm^2^	Before Therapy	41.02	16.12	12	30	44	50.5	74	0.028
		After Therapy	37.38	12.58	13	27	37	45.5	67	
	Forefoot Surface RF cm^2^	Before Therapy	46.28	17.64	17	30.5	48	58	100	0.492
		After Therapy	44.94	14.3	16	34.5	46	54	89	
	Back Foot Surface LF cm^2^	Before Therapy	39.94	9.98	17	34	39	46	60	0.015
		After Therapy	37.77	9.3	18	31	36	46	64	
	Back Foot Surface RF cm^2^	Before Therapy	40.77	8.97	23	34	42	46	58	0.975
		After Therapy	40.55	9.7	24	34	40	48.5	64	
	Load LF %	Before Therapy	48.91	8.65	25	43	48	54.5	67	0.013
		After Therapy	45.3	7.9	27	40	45	52	61	
	Load RF %	Before Therapy	51.09	8.65	33	45.5	52	57	75	0.013
		After Therapy	54.7	7.9	39	48	55	60	73	
	Max Load LF gr/cm^2^	Before Therapy	1257.1	322.85	483	1030.5	1240	1420	2063	0.913
		After Therapy	1251.5	295.9	656	1047	1191	1494	2042	
	Max Load RF gr/cm^2^	Before Therapy	1231.9	297.32	708	1017.5	1178	1428.5	2104	0.049
		After Therapy	1314.2	293.88	762	1115	1221	1592.5	1899	
	Mean Load LF gr/cm^2^	Before Therapy	528.42	139.39	241	424.5	505	598.5	902	0.733
		After Therapy	517.75	114.8	267	447	500	604	912	
	Mean Load RF gr/cm^2^	Before Therapy	511.81	124.72	276	425.5	492	565	857	0.012
		After Therapy	553.92	122.92	327	466.5	522	644	804	

**Table 4:** Analysis of the patients’ postural control parameters before and after the therapy.

Postural control parameters	Swing Length OE	Before Therapy	2354.98	730.41	1092.79	1885.21	2254.8	2774.54	4215.75	0.195
		After Therapy	2210.12	814.55	975.24	1575.07	2149.6	2633.83	5364.26	
	Swing Length CE	Before Therapy	2303.26	714.71	1076.54	1757.7	2206.74	2713.84	4516.24	0.655
		After Therapy	2351.03	836.93	860.79	1826.64	2167.19	2821.85	5172.22	
	Ellipse Surface OE	Before Therapy	745.28	1112.12	73.33	233.53	425.5	829.63	6758.89	0.009
		After Therapy	453.52	410.26	18.57	185.045	331.25	585.845	2274.39	
	Ellipse Surface CE	Before Therapy	644.76	505.76	51.74	255.085	496.41	955.96	2139.99	0.263
		After Therapy	561.28	498.75	62.23	214.27	463.39	734.865	2801.3	
	Mean Speed OE	Before Therapy	38.7	12.06	17.9	31.27	36.95	45.535	70.94	0.18
		After Therapy	36.27	13.37	15.95	25.835	35.2	43.205	88.02	
	Mean Speed CE	Before Therapy	37.87	11.71	17.67	29.535	36.25	44.475	74.13	0.687
		After Therapy	38.59	13.7	14.12	30.07	35.57	46.25	84.66	
	Max Deviation OE	Before Therapy	2.37	2.55	0.92	1.55	1.86	2.255	18.81	0.054
		After Therapy	1.86	0.78	0.95	1.42	1.73	2.085	6.24	
	Max Deviation CE	Before Therapy	2.14	1.13	1.23	1.595	1.94	2.32	8.81	0.888
		After Therapy	2.11	0.93	0.86	1.47	1.83	2.45	5.19	
	Min Deviation OE	Before Therapy	0.003	0.0046	0	0	0	0.01	0.01	0.251
		After Therapy	0.004	0.0049	0	0	0	0.01	0.01	
	Min Deviation CE	Before Therapy	0.004	0.0049	0	0	0	0.01	0.01	0.336
		After Therapy	0.0049	0.005	0	0	0	0.01	0.01	

**Table 5:** Comparison of the effect before and after the therapy with respect to dyspnoea in terms of mMRC Scale, saturation, systolic pressure, diastolic pressure, and pulse before and after the 6 min walk test [own source].

							Mean	Standard Deviation	Minimum	Q1	Median	Q3	Maximum	*p*
				
Before the 6 min walk test	DYSPNOEA mMRC	Before Therapy	After Therapy	*p*	SATURATION	Before Therapy	95.4	1.36	93	94	95	96.25	98	**0**
						After Therapy	97.26	1.58	93	96.75	98	98.25	99	
					SYSTOLIC PRESSURE	Before Therapy	133.93	15.47	97	127.75	137	143	161	0.242
	0	2.40%	11.90%	**0**		After Therapy	131.48	14.87	102	120.5	131.5	142.25	159	
	1	16.70%	71.40%		DIASTOLIC PRESSURE	Before Therapy	84.52	9.42	63	78.25	86	90.25	106	0.121
	2	57.10%	11.90%			After Therapy	82.43	10.31	59	74	81	91	106	
	3	21.40%	4.80%		PULSE	Before Therapy	77.6	11.5	51	69	80	84.5	107	**0.033**
	4	2.40%				After Therapy	73.57	7.62	52	69.75	74	80	92	
After the 6 min walk test	DYSPNOEA mMRC	Before Therapy	After Therapy	*p*	SATURATION	Before Therapy	96.57	1.19	94	96	97	98	98	0.088
						After Therapy	97.1	1.1	94	96	97	98	99	
					SYSTOLIC PRESSURE	Before Therapy	135.81	21.39	38	126.25	136.5	148	170	0.084
	0	2.40%	9.50%	**0**		After Therapy	132.88	13.39	96	125.75	133.5	140	163	
	1	16.70%	66.70%		DIASTOLIC PRESSURE	Before Therapy	83.88	7.96	67	80	84	88	110	0.172
	2	52.40%	11.90%			After Therapy	82.5	11.25	62	76.75	81	88	137	
	3	26.20%	11.90%		PULSE	Before Therapy	79.12	12.39	53	70	79.5	84.5	117	**0.046**
	4	2.40%				After Therapy	74.95	9.87	41	72	75	81	94	

## Data Availability

The data presented in this study are available on request from the corresponding author due to (The data presented in this study are made available at the request of the corresponding author due to the limitations of RODO data availability).
